# 3D Morphological Feature Quantification and Analysis of Corn Leaves

**DOI:** 10.34133/plantphenomics.0225

**Published:** 2024-09-12

**Authors:** Weiliang Wen, Jinglu Wang, Yanxin Zhao, Chuanyu Wang, Kai Liu, Bo Chen, Yuanqiao Wang, Minxiao Duan, Xinyu Guo

**Affiliations:** ^1^ Information Technology Research Center, Beijing Academy of Agriculture and Forestry Sciences, Beijing 100097, China.; ^2^ Beijing Key Lab of Digital Plant, National Engineering Research Center for Information Technology in Agriculture, Beijing 100097, China.; ^3^ Beijing Key Laboratory of Maize DNA (DeoxyriboNucleic Acid) Fingerprinting and Molecular Breeding, Maize Research Center, Beijing Academy of Agriculture and Forestry Sciences, Beijing 100097, China.

## Abstract

Marked variations in the 3-dimensional (3D) shape of corn leaves can be discerned as a function of various influences, including genetics, environmental factors, and the management of cultivation processes. However, the causes of these variations remain unclear, primarily due to the absence of quantitative methods to describe the 3D spatial morphology of leaves. To address this issue, this study acquired 3D digitized data of ear-position leaves from 478 corn inbred lines during the grain-filling stage. We propose quantitative calculation methods for 13 3D leaf shape features, such as the leaf length, 3D leaf area, leaf inclination angle, blade-included angle, blade self-twisting, blade planarity, and margin amplitude. Correlation analysis, cluster analysis, and heritability analysis were conducted among the 13 leaf traits. Leaf morphology differences among subpopulations of the inbred lines were also analyzed. The results revealed that the 3D leaf traits are capable of revealing the morphological differences among different leaf surfaces, and the genetic analysis revealed that 84.62% of the 3D phenotypic traits of ear-position leaves had a heritability greater than 0.3. However, the majority of 3D leaf shape traits were strongly affected by environmental conditions. Overall, this study quantitatively investigated 3D leaf shape in corn, providing a reliable basis for further research on the genetic regulation of corn leaf morphology and advancing the understanding of the complex interplay among crop genetics, phenotypes, and the environment.

## Introduction

The leaf constitutes a crucial structural and functional component of crops. Its morphological and structural attributes have a decisive effect on various yield and stress resistance traits in crops, including plant architecture [1], light use efficiency [2], and lodging resistance [3]. At present, research on leaves has focused primarily on easily measured indices, such as leaf length (LL), leaf area, and leaf inclination. The 3-dimensional (3D) spatial morphology of leaves is an inherent genetic trait or is influenced by environmental factors. While common sense suggests that the spatial morphology of leaves is strongly influenced by environmental factors, there is a lack of relevant research to substantiate this assertion. For example, drought and water deficiency may result in leaf wilting, whereas intense radiation can induce leaf curling. Therefore, characterizing the 3D morphological traits of leaves quantitatively presents a major challenge and a bottleneck for in-depth leaf morphology research.

Crop phenomics [4,5] has emerged as a thriving field in crop science research in recent years, driving the rapid advancement of fundamental research on the formation mechanisms of key crop traits on the basis of multi-omics approaches. The 3D crop phenotype [6,7] represents a crucial avenue for investigating the biological mechanisms underlying morphological and structure-related traits in crops. With the exponential growth of LiDAR acquisition technology [6] and multi-view 3D reconstruction techniques [8], fast, precise, and cost-effective acquisition of 3D crop data has become feasible. Furthermore, driven by the demands of biological mechanism research, researchers have significantly expanded the scope of research on crop morphological and structural traits beyond traditional manual measurement methods. At the population scale, Zhu et al. [9] utilized point cloud data acquired by backpack LiDAR to propose 3D canopy surface area, canopy spatial features, and other characteristics to elucidate the variation in wheat population structure and phenotype. Liu et al. [10] posited that the canopy occupancy volume (COV) serves as a pivotal index of canopy photosynthetic capacity. At the individual scale, Jin et al. [11] introduced 3D phenotypic traits derived from point clouds, such as PLA (projected leaf area), PAI (plant area index), and 3DPI (3D profile index), for estimating aboveground biomass. At the organ scale, Li et al. [12] extracted 77 phenotypic traits from sorghum inflorescence via computed tomography (CT) imaging, revealing the genetic diversity underlying continuous morphological variation in sorghum inflorescence.

Research on plant leaf morphological and structural phenotype analysis and 3D model construction has revealed that leaves exist mostly in a planar form [10,13], making it difficult to construct different curly and twisted leaf shapes and calculate light interception [14] or establish quantitative relationships with yield. To overcome these limitations and develop a leaf morphological structure that can capture variety- or environment-induced differences, researchers have progressively undertaken extensive research in recent years to increase the resolution of descriptions of leaf morphology. Baret et al. [15] proposed an approach to evaluate the leaf rolling of corn leaves affected by drought, utilizing bottom images of corn canopies in the field. Nevertheless, this method is limited to observing leaf differences at the population scale and is inadequate for describing morphological and structural variations at the individual or organ level. To address these shortcomings, researchers have developed applications focused on plant leaf margin diversity that can construct models of distinct leaf morphologies [16]. Zheng et al. [17] introduced a 3D modeling technique for wheat leaves driven by leaf vein curves, enabling the transplantation of leaf shape characteristics from a leaf template to other leaves with minimal energy alteration. Zhang et al. [18] proposed a modeling method for rice leaf surfaces that can simulate the morphological traits of leaf vein curvature and leaf curl (cross). Furthermore, Tross et al. [19] adopted the 3D reconstruction method of plant point clouds to identify loci linked to variations in the angle of individual sorghum leaves. Wu et al. [20] identified the 3D morphological structure-related loci of the vein curve of the ear leaf of corn through the analysis of 3D digitized data. Wen et al. [21] developed an accurate and semantic 3D reconstruction of corn leaves utilizing 3D point cloud data, achieving consistent triangular facets, and each vertex can be identified as a vein, an edge, or other vertex. However, the above studies focused mainly on the diversity of leaf margins and spatial characteristics of leaf surface profiles without considering the 3D quantitative phenotyping and morphological variation characteristics of leaf surfaces.

Currently, methods for acquiring and quantitatively characterizing the morphological traits of plant leaves in 3D space are lacking. Additionally, the mechanism behind their formation, whether it is more influenced by genetic characteristics or environmental factors, remains unclear. Furthermore, the correlations among 3D leaf shape characteristics are yet to be fully understood, and there is a lack of research approaches for ideal plant leaf shapes. To address these gaps, this paper takes the ear-position leaves of corn as a case study and proposes a quantitative 3D leaf shape calculation method. This method acquires 3D digitized data from natural populations, providing an example for the discovery of knowledge on 3D leaf shapes in plants. To further elucidate the genetic basis of 3D leaf shape in the ear leaves of corn, this study utilized an association analysis population comprising 478 inbred lines as materials. This comprehensive approach guides the selection and breeding of desired leaf shapes.

## Materials and Methods

### Field experiments

The experimental investigation was conducted at the Nanfan breeding station of the Maize Research Center, Beijing Academy of Agriculture and Forestry Sciences, which is located in Yazhou District, Sanya City, Hainan Province (longitude: 109.1832, latitude: 18.3623). The study utilized a corn association analysis population encompassing 478 inbred lines, as reported by Yang et al. [22]. These inbred lines were categorized into 4 distinct subpopulations: 122 NSS, 28 SS, 217 TST, and 111 mixed materials.

The corn was planted on 2021 March 17 with a standardized sowing density. Specifically, the row spacing was set at 60 cm, and the plant spacing was maintained at 30 cm. Each material was planted in a configuration of 2 rows by 9 plants, as detailed in the methodology of Wu et al. [20]. This experimental design allowed for a comprehensive evaluation of the diverse corn inbred lines under standardized agricultural conditions.

### 3D digitized data acquisition of ear-position leaves

**Fig. 1.**
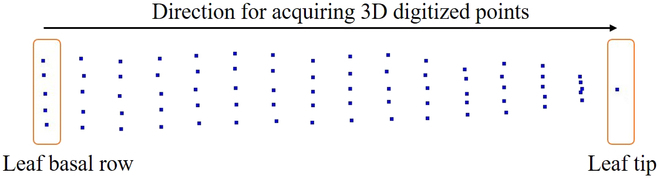
Schematic diagram of 3D digitized leaf data acquisition.

Owing to the varying growth stages of the corn population planted, the endosperm filling period differs among varieties, providing an ample time span for the data acquisition of diverse varieties. Data acquisition was carried out from 2021 May 17 to 27. When a particular variety reached the endosperm filling stage, destructive transplanting of the plant was conducted, and the plant was transported indoors for data acquisition. During transplanting, a shovel was used to excavate the root system and soil within a 25-cm diameter and 20-cm depth surrounding the plant. The plant was then placed in a pot (all sampling was performed before 10 AM) and watered after being transported indoors to minimize significant morphological changes caused by plant water deficiency [23]. The FastScan and FastRak 3D digitizers, combined with the Tx4 calibration transmitter, were employed to gather data on each plant’s ear leaf via a digital stylus [23]. The 3D coordinates of the selected leaf points were obtained one by one through manual operation (Fig. 1). The average acquisition time for each leaf was 5 to 8 min. If a plant had multiple ears, the leaf with the largest ear was selected as the ear leaf. The data were checked visually with a real-world leaf to guarantee accuracy.

To ensure universality in subsequent data processing and analytical algorithms, all ear leaves were collected under uniform data acquisition standards [24]. Using the stylus, starting from the junction of the leaf sheath and the blade, 3D feature point coordinates of the leaf shape were obtained gradually from the base to the tip of a leaf. Five points were acquired per row, with only the leaf tip point acquired in the last row (Fig. 1). If *n* + 1 rows of points were obtained for each leaf, the entire leaf comprised 5*n* + 1 points, including *n* + 1 points on the leaf vein and 2*n* points on the leaf edge. On average, the acquired data contained 16.6 rows of points, which equated to an average of 79 points (15.6 × 5 + 1) per leaf. All the acquired leaf margin feature points must include all the crease feature points present on the current leaf. Because individual leaves lack global spatial information, to provide a directional reference for leaf data, an additional point was acquired above and below the ear leaf on the stem, enabling the determination of the updown orientation of each leaf and identification of the adaxial and abaxial surfaces. To ensure the accuracy of the subsequent analyses, all the acquired data were screened, and low-quality data were excluded. The actual dataset included a total of 776 leaf samples.

### 3D leaf morphological feature extraction

**Fig. 2.**
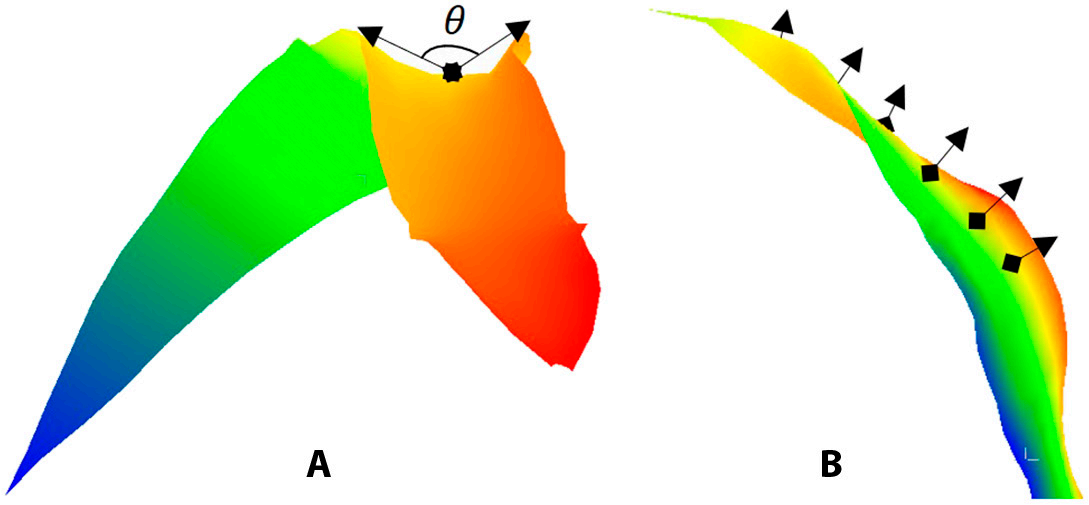
Illustration of 2 3D leaf morphological traits. (A) BIA, and (B) BST. The colors of the leaves are only used to enhance the visualization effect and increase the degree of differentiation of different parts.

To analyze the morphological differences of corn leaves in 3D space quantitatively, several corn leaf morphological parameters, such as the surface angle, self-twisting, planarity, and margin amplitude (MA) (Table 1), were proposed for quantitative description and analysis. Three parameters, namely, LL, 3D leaf area (LA), and leaf inclination angle (IA), were calculated as the overall values of the leaf surface. The calculation of the remaining 4 parameters, specifically the leaf blade-included angle (BIA) (Fig. 2A), blade self-twisting (BST) (Fig. 2B), blade planarity (BP), and MA, requires dividing the entire leaf surface into multiple segments along the leaf veins, computing the local morphological characteristics of each segment, and then obtaining the mean and standard deviation of these characteristics over the whole leaf surface as quantitative values for depicting the morphological and structural features of the leaf surface. Taking BP as an example, its mean is represented by BP_avg_, and the standard deviation is represented by BP_sd_.

**Table 1.** Overview of the extracted 3D leaf shape parameters

ID	Parameter name	Identifier	Explanation of the traits	Extended variables	Parameter type	Units
1	Leaf length	LL	Leaf vein length	-	Global feature	cm
2	3D leaf area	LA	3D leaf area	-	Global feature	cm^2^
3	Inclination angle	IA	Angle between leaf basal vein and horizontal direction	-	Global feature	°
4	Blade included angle	BIA	Angle between the 2 halves of the blade separated by the leaf vein	*BIA*_avg_, *OBIA*_avg_, *OBIA*_sd_	Local mean and standard deviation feature	°
5	Blade self-twisting	BST	Leaf blade twisting measure along the vein	*BST*, *OBST*, *OBST*_sd_	Global and standard deviation feature	-
6	Blade planarity	BP	Distance of leaf margin points to the blade	*BP*, *BP*_sd_	Global and standard deviation feature	cm
7	Margin amplitude	MA	Difference in distance between the normal projections of neighboring edge points	*MA*_avg_, *MA*_sd_	Local mean and standard deviation feature	cm

#### Calculation of LL, LA, and IA

LL was calculated as the sum of distances between adjacent vein points.

**Fig. 3.**
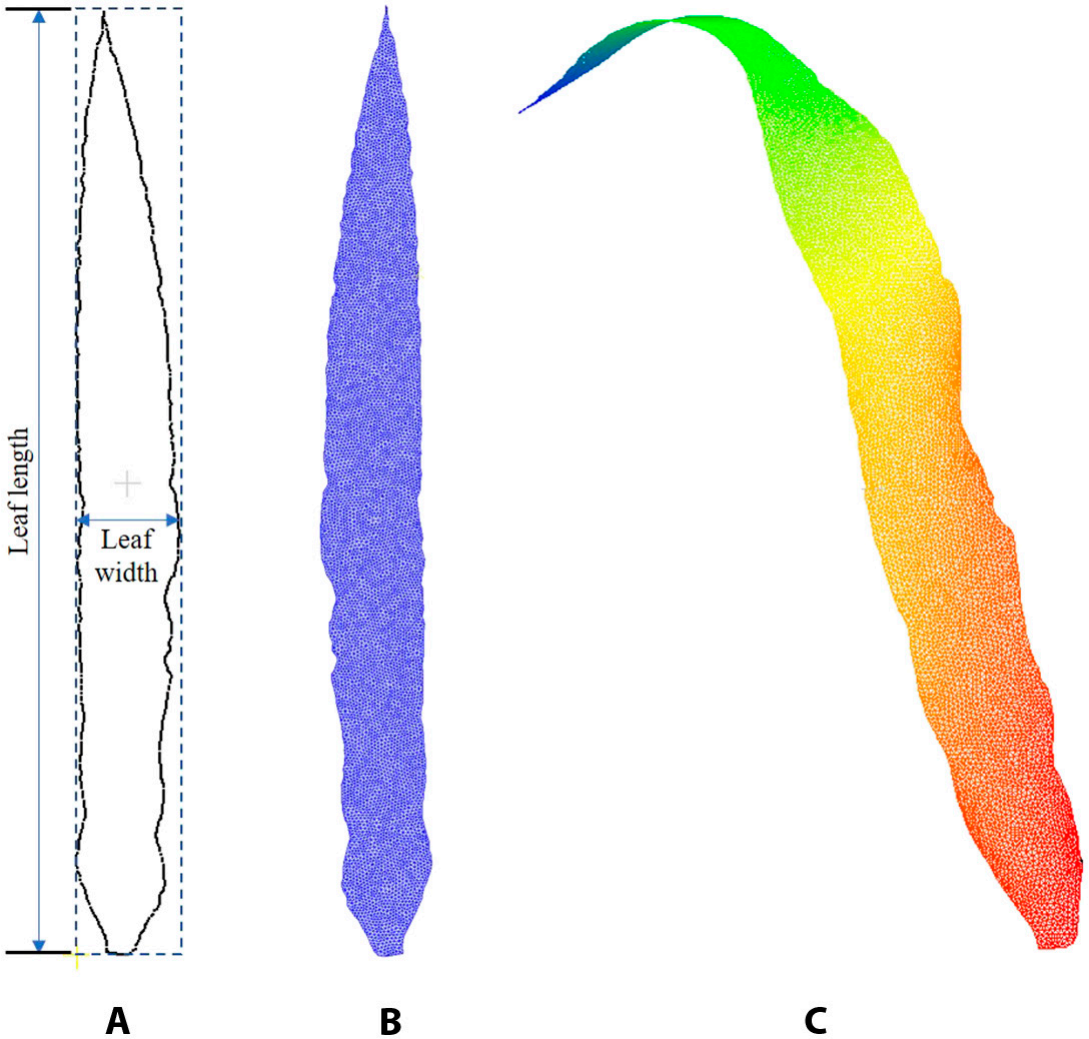
Illustration of leaf area estimation in different dimensions. (A) 1D leaf area estimation by multiplication of LL, the leaf width, and a proportional coefficient, typically 0.75. (B) 2D leaf area estimation by spreading a leaf into a flat surface [21]. (C) LA estimation by summing the 3D facet areas on a leaf. The colors in (C) are only for enhancing the visualization effect and increasing the differentiation of different parts.

**Fig. 4.**
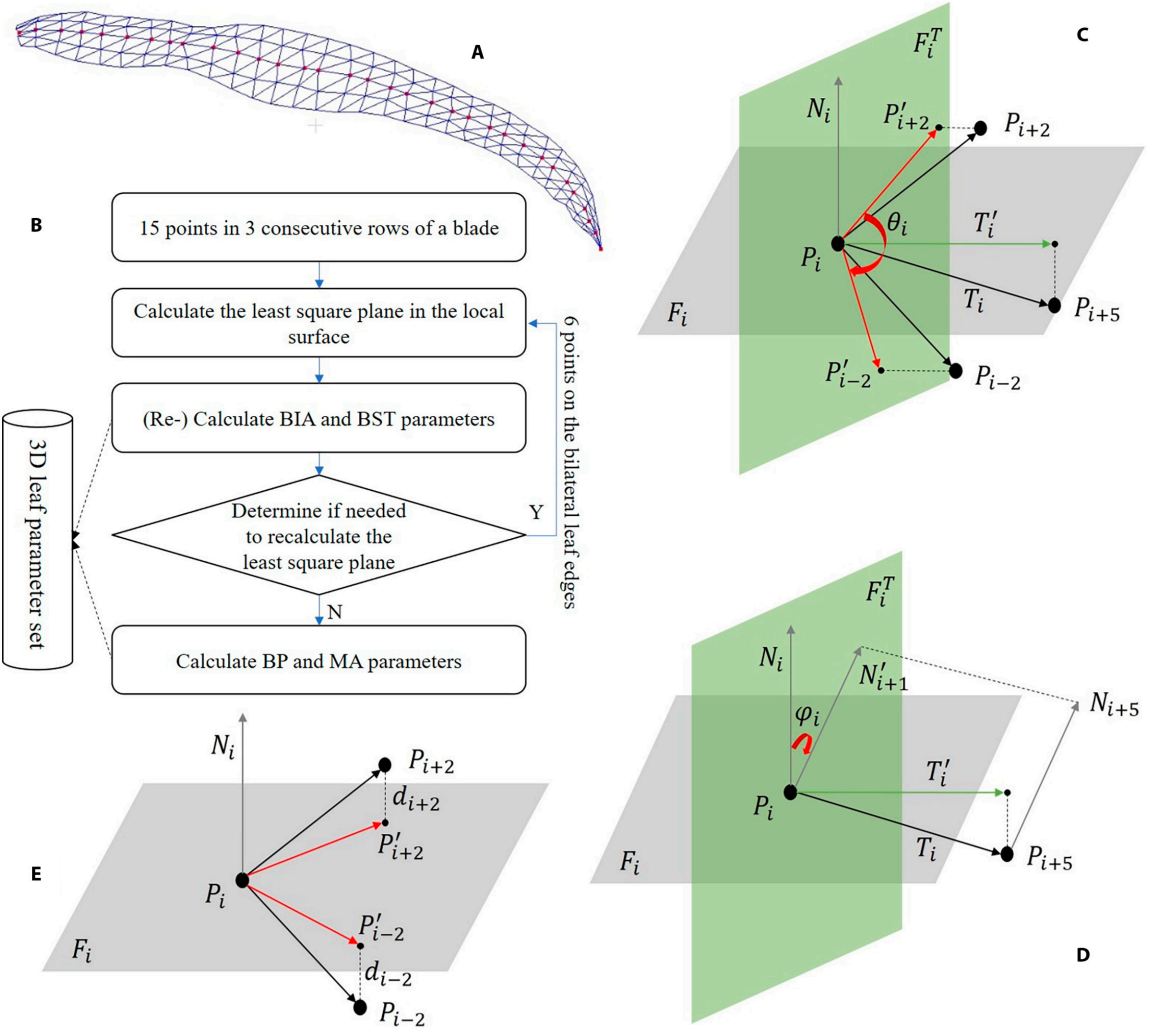
Schematic diagram of the 3D leaf shape feature parameter calculation method. (A) Illustration of the triangular mesh from digitized leaf data. (B) Flowchart of the local geometric feature extraction method for leaf blades. (C) Local BIA calculation. (D) Local BST calculation. (E) Local BP and MA calculation.

The traditional leaf area (LA) was obtained by multiplying LL by the leaf width and a proportional coefficient, typically 0.75 (Fig. 3A). However, this method often yields relatively large errors due to differences in the empirical coefficients among various leaf shapes. An improved method involves flattening the leaf and acquiring its front view image through a digital camera or flatbed scanner, resulting in a 2D leaf area measurement (Fig. 3B). This enhanced the accuracy of the leaf area calculations. Nevertheless, the presence of folds on corn leaf surfaces made it difficult to reflect differences in area on the basis of smoothness via the 2D method. In this study, 3D digitized point data were utilized to discretize the leaf surface into multiple triangular meshes. Specifically, vertices in the same row and vertices in adjacent rows were connected to form triangular meshes (Fig. 4A). The area of each triangular facet was computed, and the sum of these areas provided the LA of the current leaf surface (Fig. 3C). Compared with 1D and 2D methods, this approach offers a more accurate means of describing leaf area, as it takes into account area differences caused by morphological characteristics, such as folding and bending.

IA was determined by calculating the angle between the venation curve near the base of the leaf and its projection onto the horizontal plane.

#### Calculation of the local geometric features of corn leaves

The shape of the corn leaves exhibited considerable variation, yet local areas on the surface can be simplified to form planes that hold vital local geometric features. These planes are fundamental for calculating 3D leaf shape parameters. Hence, it was imperative to break down the surface of a leaf into several local planes and determine the direction of each plane’s normal vector. Through the 3D digitization approach, a leaf blade was locally broken down. For a leaf blade with *n* rows of points, 3 consecutive rows make up a local leaf surface. This forms *n* − 2 local leaf surfaces, excluding the first and last rows, as they are at the edge and are not considered leaf surfaces. Each local leaf surface consists of 15 points, with the mid-row vein point being the central point. The point set-based plane approximation method in the VCG library (Visualization and Computer Graphics Library) was used to estimate each locality of the leaf surface. The normal direction of the approximating plane was regarded as the normal direction of the local leaf surface. By following this procedure, direction information for each leaf surface’s local plane was gathered.

Owing to the high complexity of leaf morphology in corn inbred lines, such as leaves with large BIAs or BSBs, obtaining accurate local leaf normal information via the above method is not possible. As a result, for leaf shape features with erroneous calculations, the local normal was estimated via bilateral leaf edge data points with local leaf shape parameters as a reference (Fig. 4B). If the 3D measurements of leaf shape, obtained via 15 points on the local leaf surface, satisfy one of the prescribed threshold discrimination conditions, then the local geometric properties of the leaf will be estimated via the bilateral leaf edge points on the local leaf surface.

$${\mathrm{BST}}_{\mathrm{avg}}>r_{1}\;\text{or}\;\left|{\text{OBIA}}_{\mathrm{avg}}-180.0\right|>r_{2}\;\text{or}\;{\text{OBIA}}_{\mathrm{sd}}>r_{3}$$ (1)

In the previous formula, *r*_1_, *r*_2_, and *r*_3_ were the discrimination thresholds. The thresholds were determined through a process of checking and statistical analysis of the leaf samples, which revealed an estimated normal on the local leaf surface that was deemed incorrect. For practical calculations, *r*_1_ = 1.5, *r*_2_ = 50.0, and *r*_3_ = 65.0 were used.

#### Calculation of BIA

BIA quantifies the angle formed by 2 halves of a corn leaf blade, indicating the degree of inner rolling, flattening, or outer rolling. This morphological response in leaves is a significant indicator of water stress or intensified radiation conditions.

To explain how the BIA-related parameters are calculated, we take the computation of the local BIA (*θ_i_*) at the central point (*P_i_*) as an example. As shown in Fig. 4C, *P_i_* is the central point of the local leaf blade, which corresponds to a vein point. On the same row of *P_i_*, *P*_*i*−2_ and *P*_*i*+2_ are the margin points, whereas *P*_*i*+5_ denote the vein point on the subsequent row. *N_i_* denotes the normal vector of the local plane (*F_i_*) calculated via the local geometric feature calculation method. *T_i_*^′^ represents the projection vector of vector *T_i_* = *P*_*i*+5_ − *P_i_* onto the plane (*F_i_^T^*) with *N_i_* as its normal vector. The projection points (*P*^′^_*i*−2_ and *P*^′^_*i+*2_) of *P*_*i*−2_ and *P*_*i*+2_ onto the plane (*F_i_^T^*) were calculated, and the angle ∠*P*^′^_*i*−2_*P_i_ P*^′^_*i+*2_ on the plane (*F_i_^T^*) constituted the present local BIA, referred to as *θ_i_*.

The local BIA calculation method indicated that 0° < *θ_i_* < 360°. To prevent angles less than 180°, the positive direction of the blade normal direction at the leaf base was determined according to the plant’s growth direction. Additionally, the angle between neighboring local blade normals should be limited to 180° on the basis of the continuity of adjacent local leaf surfaces. The calculation of the local BIAs was facilitated by determining the positive direction of all the blade normals, enabling a clear distinction between the upper and lower angles. The angle exhibited directionality in accordance with established conventions. To represent the oriented BIA (OBIA) of a leaf blade with *n* rows of points, the average OBIA (*OBIA*_avg_) of its *n* − 2 local blade angles was used. *OBIA*_avg_ ranged from 0° to 360°.

Furthermore, to investigate the magnitude of the leaf blade angle, disregarding directional features, the range of each local BIA was constrained to (0°, 180°] on the basis of OBIA. Specifically, when *θ_i_* > 180°, *θ_i_*′ = 360° − *θ_i_*; conversely, when *θ_i_* ≤ 180°, *θ_i_*′ = *θ_i_*. This approach defines the average nondirectional leaf blade angle of the *n* − 2 local leaf blades as the current BIA, denoted as *BIA*_avg_∈(0°, 180°].

Certain leaf varieties exhibit segmented disparities in leaf BIAs. Consequently, we introduced the standard deviation of OBIAs (*OBIA*_sd_) to characterize these variations quantitatively. *OBIA*_sd_ was calculated via the *θ_i_* values of the *n* − 2 local leaf surfaces. A higher value of *OBIA*_sd_ signified greater segmented variation in the overall BIAs across the entire leaf. This metric enables a more comprehensive and quantitative assessment of BIA variations across different leaf varieties.

#### Calculation of BST

BST was used to characterize the local and global directional variation in leaves from base to tip, which was also estimated through discrete local leaf geometric features. As depicted in Fig. 4D, *P_i_* denotes the central point of the local leaf surface, *F_i_* represents the approximated plane of the local leaf surface, and *N_i_* represents its normal vector. *P*_*i*+5_ corresponds to the next row of vein points, *N*_*i*+5_ is the associated local leaf surface-approximated plane normal vector, and *T_i_*^′^ indicates the projection vector of *T_i_* = *P*_*i*+5_ − *P_i_* on the plane *F_i_^T^* along the normal vector *N_i_*. The projection vector *N*^′^_*i*+1_ of vector *N*_*i*+5_ on the plane *F_i_^T^* passing through point *P_i_* and normal to *T_i_*^′^ was computed. The angle between vectors *N_i_* and *N*^′^_*i*+1_ on the plane *F_i_^T^* constituted the local self-twisting angle of the current local leaf surface, designated *φ_i_*. Notably, the local self-twisting angle demonstrated directionality, with *N_i_* rotating counterclockwise to *N*^′^_*i*+1_ defined as positive and clockwise rotation defined as negative. If a blade comprises *n* rows of points, the *n* − 2 local leaf surface of this blade yields *n* − 3 local self-twisting angles. By employing these *n* − 3 self-twisting angles and calculating them according to Eq. (2), the directional BST of the current blade was obtained, denoted as *OBST*, representing the global feature of the blade. For example, if the BST was 1.2, the blade twisted by 1.2 times 180°, which is equivalent to 216°. This metric facilitates a comprehensive understanding of the twisting behavior of leaf blades and provides insights into their complex geometric features.

$$\text{OBST}=\left|\sum_{i=1}^{n-3}\varphi_{i}\right|/180.0$$ (2)

Similarly, to study BST without considering directional characteristics, BST was defined as *BST* = |*OBST*|. Furthermore, the local BST angle exhibited segmented variations throughout the entire blade. To quantitatively describe these variations, the directional standard deviation of BST, denoted as *OBST*_sd_, was introduced. *OBST*_sd_ was calculated via the aforementioned *n* − 3 local self-twisting angles. The results showed that a larger *OBST*_sd_ indicated greater segmented variability in the BST angle across the entire blade. This finding provides a more comprehensive understanding of BST and its variations.

#### Calculation of BP and MA

Corn leaves exhibit varying 3D morphological characteristics attributed to the count, distribution, and dimensions of folds. For quantitative assessment of these features, BP and MA were introduced. Specifically, the calculation methods for BP and MA were exemplified via the local leaf surface centered at point *P_i_*. As illustrated in Fig. 4E, *P_i_* denotes the central point of the local leaf surface, which is also a point on the leaf vein. *P*_*i*−2_ and *P*_*i*+2_ represent the edge points of the same row as *P_i_*, whereas *N_i_* signifies the normal vector of the current local leaf surface plane *F_i_* passing through point *P_i_*. The projection points *P*^′^_*i*−2_ and *P*^′^_*i+*2_ of the edge points *P*_*i*−2_ and *P*_*i*+2_ on plane *F_i_* were calculated. The distance from the edge point to the local approximated plane was defined as *d*_*i*+2_ = |*P*^′^_*i+*2_- *P*_*i*+2_|. Because the edge points could be located on either side of the local approximated plane, when the edge point was on the upper side of the local plane, i.e., on the same side as *N_i_*, the distance value was positive, and vice versa. For a leaf with *n* − 2 local leaf surfaces, there are 2 (*n* − 2) edge points. Accordingly, the calculation method for BP is defined as follows:

$$\mathrm{BP}=\sum_{i=1}^{2\left(n-2\right)}\left|d_{i}\right|/\left(2n-4\right)$$ (3)

Similarly, BP is characterized by piecewise dissimilarity. To capture this attribute, the standard deviation of BP (*BP*_sd_) was introduced. This metric was calculated via the abovementioned 2(*n* − 2) absolute distance values.

Moreover, the fluctuation in the corn leaf edge curve constituted a pivotal feature of the 3D leaf shape. This aspect was quantified by the leaf MA. When the distance (*d_i_*) between the *n* − 2 blade edges and the blade surface computed via the same-side blade edge mentioned earlier is considered, the absolute difference between adjacent distances represents the amplitude of the local blade edge. The mean value of the entire MA (*MA*_avg_) was subsequently calculated as follows:

$${\mathrm{MA}}_{\mathrm{avg}}=\left(\sum_{i=1}^{n-3}\left|d_{i+1}-d_{i}\right|+\sum_{j=1}^{n-3}\left|d_{j+1}-d_{j}\right|\right)/\left(2n-6\right)$$ (4)

where *d_i_* and *d_j_* represent the distances between the edges of the 2 sides of the blade and the local leaf surface, respectively.

The segmented difference in the blade MAs was also described by *MA*_sd_.

### Mesh generation and data visualization

The parameters for calculating the 3D shape of a leaf depend on the coordinates of the points within the 3D digitized data. However, visualizing the shape of a leaf with sparse 3D point clouds poses a challenge. To overcome this issue, a blade mesh was generated and visualized via digitized 3D data. Adjacent points in each row and column were joined to form a quadrilateral mesh. These meshes were then split into 2 triangles, and the blade tips were directly connected to form triangles. A triangular mesh model of the entire blade was generated. However, the model generated was not smooth enough, prompting the use of the Sqrt3 mesh subdivision method [25] to refine it. This resulted in a smoother mesh model, which was used to visualize the leaf meshes in Figs. 3 and 4. The blades were also rotated to enhance the presentation of the leaf shape characteristics, so the visualized mesh seemingly does not match the actual leaf inclination. The visualization of each blade depicts 2 views, with blue indicating the front of the blade and yellow indicating the back.

### Statistical analysis of phenotypic data

R software (version 3.6.3) was used for descriptive statistical analysis of the phenotypic data distribution derived from the above 3D leaf feature extraction. The Spearman rank correlation coefficient method was used to assess the relationships between traits. To ensure accurate results, the Holm correction was implemented for multiple tests [26]. Furthermore, to identify inherent patterns and relationships within complex datasets, cluster analysis was utilized in this study. The phenotypic data were clustered after being standardized via Z scores, and visualization was achieved via the R package ComplexHeatmap.

To further elucidate the classification of 3D leaf shape types, K-medoid clustering was performed via the pam function from the R package cluster. The optimal K was selected as the final number of clusters, and a scatterplot of the samples after MDS classification was generated via ggplot2. Intergroup difference analysis was subsequently conducted on the classified sample subsets.

To compare the similarities and differences among different genetic background materials, specifically between different subgroups, we conducted trait network analysis via the R package NetComi on tropical materials (TST) and temperate materials (SS, NSS, and Mixed) and compared the differences between the networks. Furthermore, analysis of variance (ANOVA) was conducted to evaluate differences in traits among subpopulations, and the Holm method [26] was applied to correct for multiple tests. Differences in traits were illustrated via radar charts to highlight distinguishing features among subpopulations.

In ASReml-R version 4.0, the generalized heritability (*H^2^*) of 13 phenotypic traits pertaining to ear leaves was estimated. This was achieved by utilizing the “asreml” function of the R software package asreml. The estimation procedure was performed as follows:

$$H^{2}=\frac{V_{g}}{V_{g}+\frac{V_{\mathrm{GL}}}{L}+\frac{V_{e}}{L*R}}$$ (5)

where *L* represents the number of locations; *R* denotes the number of repetitions; and *V_g_*, *V_GL_*, and *V_e_* correspond to the genotype variance, genotype-by-location interaction variance, and environment variance, respectively.

## Results

### 3D leaf shape feature distribution and typical leaf visualization

Leaves exhibiting typical characteristics were selected for visualization (Figs. 3 and 4). The visualization results for each leaf blade were provided from 2 perspectives, with blue indicating the front and yellow indicating the back. Every parameter displayed a normal distribution. In Table 2, the range and mean values of each leaf shape parameter are provided.

**Table 2.** Distribution ranges and mean values of various feature parameters

Trait	Mean	Range
LL	69.249	[41.290, 100.754]
LA	502.454	[193.307, 880.375]
IA	49.475	[0.650, 89.474]
*BIA*_avg_	139.609	[58.797, 170.386]
*OBIA*_avg_	173.899	[91.081, 301.203]
*OBIA*_sd_	39.352	[8.522, 90.795]
*BST*	0.289	[0.000, 1.399]
*OBST*	0.196	[−1.147, 1.399]
*OBST*_sd_	12.224	[2.836, 3.299]
*BP*	1.316	[0.425, 3.299]
*BP*_sd_	0.884	[0.315, 1.774]
*MA*_avg_	1.135	[0.270, 2.940]
*MA*_sd_	0.839	[0.238, 2.162]

**Fig. 5.**
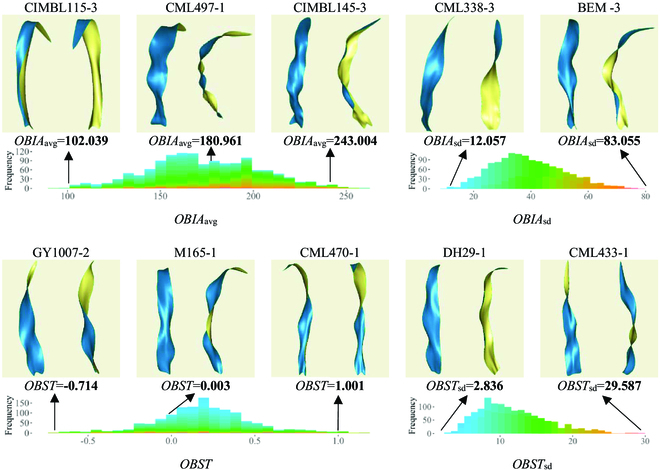
Visualization and parameter distribution of blades with typical OBIA and OBST values.

BIA describes the angle between the 2 halves of the leaf blade separated by the veins, whereas OBIA is used to characterize whether the leaf surface is significantly inner or outer rolling. In Fig. 5, the OBIA value of CML497-1 was close to 180°, suggesting that the leaf blade was spreading and that the leaf blade on both sides of the veins was neither significantly inner nor outer rolling. The OBIA value of CIMBL115-3 was smaller, suggesting an inner rolling state, whereas the OBIA value of CIMBL145-3 was larger, suggesting an outer rolling effect. OBIA_sd_ describes the overall variation in BIA from the leaf base to the leaf tip. For example, the OBIA_sd_ value of CML338-3 was small, and its visualization also showed minimal variation in the overall BIA. In contrast, the OBIA_sd_ value of BEM-3 was large, and the visualization results revealed that this leaf exhibited an outer rolling state near the leaf base, an inner rolling state in the middle of the leaf blade, and unfolding near the leaf tip, demonstrating significant variation in the overall leaf blade angle.

The BST characterizes the degree of twisting along the leaf veins on the leaf blade. OBST_sd_ depicted whether this twisting of the leaf blade was toward the left or right. As shown in Fig. 5, the BST value of M165-1 was close to zero, which indicated that the leaf surface was untwisted. Conversely, the OBST value of GY1007-2 registered a negative value, indicating a leftward twist on the leaf blade. On the other hand, the OBST value of CML470-1 was relatively high, suggesting a rightward twist. Notably, the twisting of the leaf blade was not consistently distributed across the entire leaf. OBST_sd_ quantified the heterogeneity in leaf twisting across the entire leaf surface. As an example, DH29-1 demonstrated minimal variation in twisting across its surface, resulting in a lower OBST_sd_ value. In contrast, CML433-1 did not exhibit considerable twisting at the base, but it underwent appreciable deflection in the middle to tip section, thus contributing to greater overall variation in leaf twisting.

**Fig. 6.**
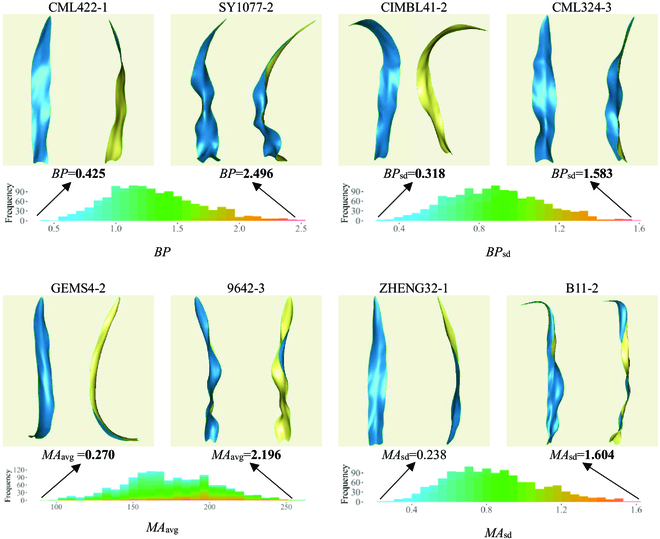
Visualization and comparative parameter distributions of leaf blades with typical BP and MA values.

The leaf surface evenness characterizes the average deviation of all the leaf margin points from the leaf surface, which is denoted as BP. A greater BP indicated a rougher leaf surface, as exemplified by SY1077-2 in Fig. 6. Conversely, a smaller BP signified a smoother leaf surface, as exemplified by CML422-1 in Fig. 6. The evenness of the leaf surface also exhibited segmented variation, which was described via the BP_sd_ value. For example, CIMBL41-2 demonstrated overall low variation in leaf surface evenness. However, in CML324-3, the portion near the leaf base was particularly uneven, whereas the middle-to-tip section of the leaf was relatively smoother, resulting in greater overall variation in leaf surface evenness and, consequently, a larger BP_sd_ value.

Figure 6 provides a visualization of leaves with both high and low average leaf MAs (MA_avg_). For example, GEMS4-2 exhibited a relatively smooth and minimally fluctuating leaf margin curve, resulting in a lower MA_avg_ value. Conversely, 9642-3 displayed significant fluctuations along its leaf margin, leading to a higher MA_avg_ value. The amplitude of the leaf margin also demonstrated segmented variation. An example of this was ZHENG32-1, which showed minimal variation along the entire leaf margin, resulting in a lower MA_sd_ value. However, in leaf B11-2, the lower to middle portions of the leaf margin exhibited substantial fluctuations, whereas the margin near the leaf tip underwent relatively minor changes. Consequently, the overall variation in the leaf MA was greater in these leaves.

### 3D leaf shape feature correlation analysis results

**Fig. 7.**
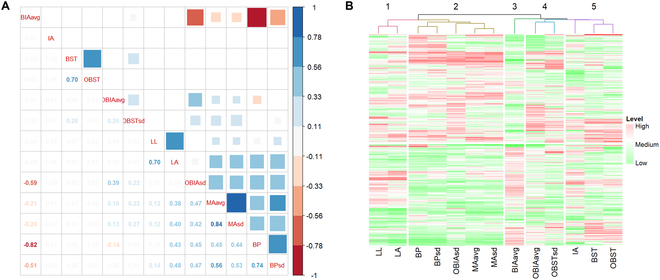
Correlation analysis and clustering results for 13 3D leaf morphological traits. (A) Correlation analysis of 13 traits. (B) Clustering results of 13 traits on the basis of normalized data.

The 3D morphological characteristics of the leaves were significantly correlated. The results of the correlation analyses of the 13 phenotypic traits studied are presented in Fig. 7A. The figure provides insights into the analysis and results obtained. The leaf area (LA) and LL were positively correlated (*r* = 0.70, *P*_adj_ < 0.05). However, the correlations between the leaf angle (IA) and other traits did not demonstrate significance after multiple testing correction. Additionally, LA was significantly positively correlated with BP and MA (*r* ranging from 0.38 to 0.48, *P*_adj_ < 0.05), and the study revealed that leaves with a greater LA were more likely to exhibit unevenness and folds at the margin. BST-related traits were observed only within certain groups and were specifically correlated with the OBST and OBST_sd_ values but not with other traits. These findings suggest that leaf twisting is not affected by factors, such as leaf rolling, smoothness, or area. The BP and MA traits were closely correlated, likely because of the association between increased margin folds and unevenness of the leaf surface. Additionally, our findings indicated that the OBIA_sd_ value exhibited a positive correlation with BP and MA, suggesting that inner and outer rolling variation contributed to greater leaf unevenness and margin folds, which is in line with our intuitive understanding. BIA_avg_ was significantly negatively correlated with both the BP (*r* = −0.82, *P*_adj_ < 0.05) and BP_sd_ (*r* = −0.51, *P*_adj_ < 0.05) values. However, OBIA has the potential to mitigate or eliminate this negative correlation, underscoring the importance of the directional leaf curling parameter.

We further processed the phenotypic data via the Z score normalization method to ensure comparability across different indicators. On the basis of the normalized data, we applied the *k*-means clustering algorithm to categorize the indicators. When the number of clusters (*k*) was set to 5, the traits BP, BP_sd_, OBIA_sd_, MA_avg_, and MA_sd_ clearly grouped together (Fig. 7B). This clustering outcome aligns closely with the previous findings from the correlation analysis of the indicators, suggesting a strong correlation among these specific metrics. Therefore, they can potentially be considered composite measures for subsequent analysis, providing a more holistic perspective on the phenotypic variations under investigation.

### Diversity and distinctive characteristics of 3D leaf morphology in corn

**Fig. 8.**
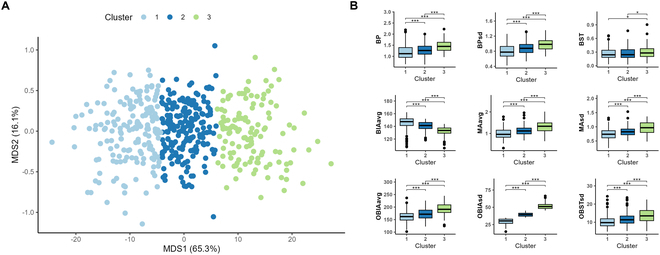
K-medoid clustering of morphological traits and differential leaf characteristic analysis in corn inbred lines. (A) K-medoid clustering results for 478 corn inbred lines on the basis of 5 key morphological traits. (B) Differential analysis of leaf morphological characteristics among the 3 clusters.

On the basis of previous correlation and clustering analyses of phenotypic traits, we conducted an in-depth investigation into the 3D leaf morphological diversity of corn, focusing on 5 key traits, namely, BP, BP_sd_, OBIA_sd_, MA_avg_, and MA_sd_. By utilizing multidimensional scaling (MDS), we successfully reduced the dimensionality of the distance matrix constructed between pairs of leaf shapes, thereby deriving new indices that effectively capture the characteristics of 3D leaf morphology. Notably, the 2 MDS axes accounted for a substantial 81.4% of the variance (Fig. 8A). To further elucidate the classification features of corn 3D leaf morphology, we applied the k-medoid clustering method to the 3D leaf morphological data of 478 corn inbred lines. By employing gap statistics, we determined the optimal number of clusters to be 3. This allowed us to categorize 3D leaf morphology into 3 distinct types.

We subsequently conducted a differential analysis of leaf morphological characteristics among these 3 inbred line clusters. The results revealed significant differences across the 9 traits (Fig. 8B). Specifically, with the exception of BIA_avg_, where cluster 3 presented the smallest values and cluster 1 the largest values, all other indices presented the highest values in cluster 3 and the lowest values in cluster 1. This finding reinforces the positive correlation between BP and MA, indicating that leaves with greater BP tend to have larger MAs. These findings provide valuable insights into the diversity of corn 3D leaf morphology and its relationship with phenotypic traits.

### Results of heritability analysis

**Fig. 9.**
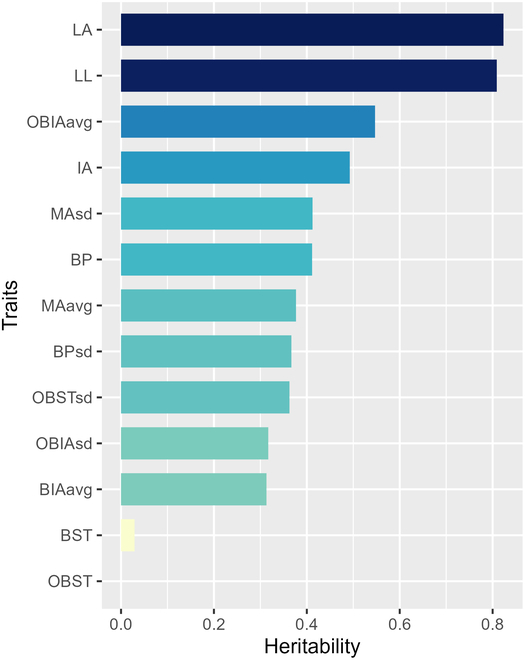
Results of broad-sense heritability analysis for 13 3D leaf phenotypic traits.

An analysis of heritability was conducted on 13 phenotypic traits of ear-position leaves in corn. The results revealed that the range of genetic heritability varied from 3.89 × 10^−7^ to 0.8233 (Fig. 9). Notably, LA and LL presented high heritability (*H*^2^ > 0.80), indicating that further exploration of their genetic mechanisms is worthwhile. In contrast, both BST and OBST demonstrated minimal heritability (close to 0) in this study, suggesting that these traits and their associated phenotypes are influenced primarily by environmental factors. However, when the overall results were considered, 84.62% (11/13) of the leaf traits presented a heritability greater than 0.3, indicating that genetic factors still play a significant role in the formation of 3D phenotypes in the ear leaves of corn.

### Analysis of differences among subpopulations

**Fig. 10.**
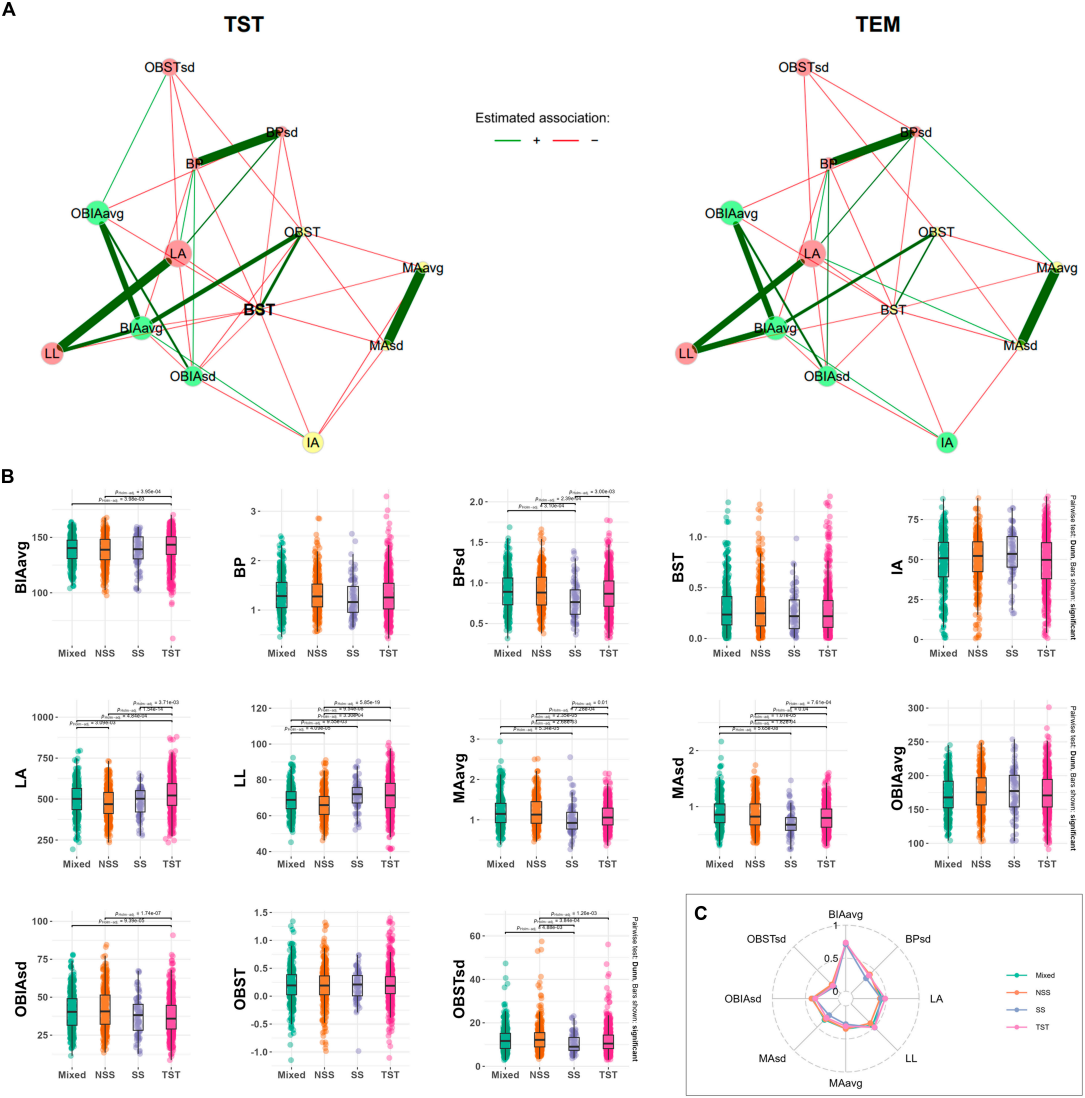
Analysis of differences in 13 3D leaf phenotypic traits between subpopulations. (A) Network analysis of 13 traits in different climatic regions (TSTs: including the TST subpopulation; TEMs: including the SS, NSS, and mixed subpopulations). Nodes represent traits, with different colors indicating various groupings. The size of the nodes signifies their importance within the network, with larger nodes denoting greater importance. The thickness of the edges signifies the closeness of the connection between traits: Thicker edges denote stronger correlations, whereas thinner edges represent weaker ones. The green edges symbolize positive correlations, whereas the red edges indicate negative correlations. (B) Analysis of differences in 13 traits between the 4 subpopulations. (C) Radar chart demonstrating differential characteristics among subpopulations of 3D corn leaf morphology.

To further investigate the variations in the 13 traits and their interactions across different climate regions, we classified the 4 subpopulations into 2 distinct categories: TST, comprising tropical inbred lines (including the TST subpopulation), and TEM, encompassing temperate inbred lines (including the SS, NSS, and mixed subpopulations). Using the R package “NetCoMi” to construct networks and conduct a comparative analysis, we first observed a consistent pattern: Regardless of their origin in diverse climate regions, the materials uniformly exhibited BST as the hub trait in terms of 3D leaf morphology. This uniformity was further underscored by the generally consistent phenotypic correlations, evident from the presence and thickness of edges within the network. However, despite these similarities, notable differences also emerged. Specifically, within the TST category, a direct correlation was observed between the OBST_sd_ and OBIA_avg_ values, a relationship that was absent within the TEM category. Furthermore, within the TST group, IA exhibited a strong association with MA_sd_, MA_avg_, OBST, and BST (highlighted by yellow nodes in Fig. 10A). However, within the TEM category, the IA values were clustered with the OBIA_sd_, BIA_avg_, and OBIA_avg_ values (indicated by the green nodes in Fig. 10A). These findings emphasize the existence of subtle but significant phenotypic variations among materials originating from distinct climate regions.

Furthermore, an analysis of 13 phenotypic traits among the 4 subpopulations was conducted. The results (Fig. 10B) revealed significant differences among the subpopulations for 8 of the traits, with particularly notable differences between the TST subpopulation and the other subpopulations. Compared with the other subpopulations, the TST inbred lines presented a significantly greater leaf area (LA) (*P*_adj_ < 0.05). Additionally, significant differences in leaf MA (MA_avg_ and MA_sd_) were detected between the TST subpopulation and the other populations (*P*_adj_ < 0.05). Interestingly, most of the traits that showed differences among subpopulations were standard deviations, indicating that segmented variations in different leaf traits exist among the subpopulations. However, when overall metrics were considered, no significant differences were observed in the IA, BP, BST, OBIA_avg_, or OBST values among the subpopulations. The radar chart (Fig. 10C) provides a more intuitive display of the differences in these 8 traits among the 4 subpopulations, with distinct characteristics particularly evident in the TST subpopulation. In conclusion, these results suggest that while overall 3D leaf morphology may not differ substantially among corn inbred lines, local variations can be more reflective of variety characteristics. Therefore, the segmented variation metrics of 3D leaf phenotypes proposed in this study can be utilized for the phenotypic characterization of tropical/subtropical or temperate inbred lines.

## Discussion

### 3D leaf shape quantification offers new perspectives for related research

LL, leaf width, IA (1D traits), and leaf area (2D trait) are crucial morphological indicators that corn breeders and cultivators prioritize. These parameters can be conveniently assessed through various tools, such as rulers, protractors, and leaf area meters, and therefore qualify as observable phenotypes. However, some leaf morphological traits are clearly noticeable but difficult to quantify directly. Other features, such as the number of leaf folds, extent of leaf rolling (either inner or outer), and degree of leaf twisting, must be considered, which can be viewed as computational phenotypes. To determine these features, the utilization of 3D spatial data (such as 3D point clouds) is indispensable. This paper outlines the techniques used to quantitatively calculate 3D parameters of leaf shape in corn leaves, including LA, BIA, BST, BP, and MA. These methods provide a strong foundation for further research in this field.

The evaluation of both 1D and 2D corn leaf traits can be performed by altering the leaf morphology. For example, LL is typically assessed through straightening the leaf, whereas leaf area is determined via a leaf area meter after the flattening of the leaf. However, it is not possible to obtain 3D metrics of corn leaves through the aforementioned morphological alterations. This limitation is demonstrated by previous studies conducted on corn leaf vein curves [20]. If the leaves are elongated, it will lead to alterations in the curvature of the leaf veins and consequently affect a set of indicators. The 3D indicators of leaf shape recommended in this study also share a similar pattern, and they more precisely portray the growth status of leaves in their native field circumstances.

Quantifying the characteristics of leaf rolling, twisting, and folding has provided a relatively high-resolution approach for quantitatively analyzing the photosynthetic efficiency of various corn varieties [27]. This method shifts the focus and spatial resolution from plant architecture to 3D leaf shape. Moreover, researchers have examined the response mechanisms of corn leaf morphology to various stress conditions, such as drought or high temperature, by quantifying leaf rolling. Additionally, quantifying BP and MA facilitated the study of photosynthetically active areas in distinct varieties. This facilitated an investigation into the traction force produced by leaf movements to resist lodging under different wind conditions. This technique can serve as a guideline for quantitative research on the morphology and structure of other crop leaves.

### Genetic and environmental factors both affect 3D leaf shape

In light of our heritability analysis, the 3D leaf morphology of corn varieties has been found to be under the influence of both genetic and environmental factors. More specifically, leaf shape features, such as LA, LL, OBIA, and IA, exhibit significant control over genetic factors with a high level of heritability. Conversely, BST and OBST demonstrate greater susceptibility to environmental factors, thereby exhibiting low heritability. These findings are in accordance with those of previous studies, which revealed relatively high levels of phenotypic heritability in terms of LL [28,29], leaf width [28–30], and leaf angle [29,31–38], with minimal variance across diverse environments. This further underscores the notable influence of genetic factors on these leaf shape traits. Given that corn leaves are exposed as thin pieces to the environment, they engage in constant interactions with environmental elements, such as light, air currents, and moisture. Any alterations in these factors can impact traits leaf flatness, folding, curling, and twisting, potentially leading to water loss, wilting, prolonged wind disturbance, and competition for light resources with neighboring plants. Hence, it is not surprising that leaf morphology is susceptible to environmental fluctuations, explaining the varying impacts of the environment on different 3D leaf shapes. Notably, our study revealed that 84.62% (11 of 13) of the 13 examined 3D phenotypic traits of ear-position leaves presented a heritability greater than 0.3, highlighting the pivotal role of genetic factors in the formation of 3D phenotypes in corn. These findings underscore the imperative need for further genetic dissection of these highly heritable 3D leaf shape phenotypes to elucidate the underlying genetic architecture and identify potential candidate genes. Such insights would provide invaluable leverage in understanding the basis of leaf shape variation and its implications for plant performance across diverse environmental gradients. However, owing to the overwhelming workload of obtaining 3D semantic leaf data, it is not feasible to obtain 3D leaf data in different environmental setups. Once more efficient information acquisition methods have been developed, it will be possible to conduct relevant research.

### Future work

Acquiring 3D corn leaf characteristics via 3D digitization methods is a time-consuming and labor-intensive process with inefficiencies. Furthermore, the extended data acquisition time may amplify the influence of environmental factors on 3D leaf morphology. Consequently, moving beyond these techniques and establishing a semantic 3D reconstruction approach based on 3D point clouds for corn leaves is crucial [21]. The acquisition of high-precision 3D point clouds is a prerequisite for 3D semantic reconstruction[39], with the acquisition time estimated at approximately 3 min per leaf. This is a relatively efficient process compared with digitized data acquisition, which typically takes between 5 and 8 min. Nevertheless, 3D semantic reconstruction is more susceptible to the absence of a point cloud, and it is challenging to achieve reconstruction of leaves with a large BIA. Nevertheless, this method offers a high degree of data quality, accuracy, and reliability and is the primary approach for subsequent 3D leaf shape research. The integration of high-throughput acquisition of 3D plant data [8] and plant point cloud segmentation techniques [40] can increase the efficiency and automation level of data acquisition processes. Notably, significant morphological variations exist among all the leaves on a plant, although this study focused primarily on ear leaves. Thus, attaining semantic 3D reconstruction of corn leaves would not only increase the efficiency of 3D leaf shape calculations but also allow for extensive 3D leaf shape examination at other leaf positions throughout the plant. This development would greatly contribute to a wider comprehension of plant morphology and its genetic and environmental influences. In fact, the quantitative impact of the environment should indeed be considered, but owing to the excessive workload of obtaining 3D semantic leaf data, it is not possible to obtain 3D leaf data in different environment setups. After more efficient information acquisition methods are available, relevant research can be carried out.

## Data Availability

The data will be made available upon request.
